# Novel 1,3,4-Oxadiazole Induces Anticancer Activity by Targeting NF-κB in Hepatocellular Carcinoma Cells

**DOI:** 10.3389/fonc.2018.00042

**Published:** 2018-03-19

**Authors:** Chakrabhavi Dhananjaya Mohan, Nirvanappa C. Anilkumar, Shobith Rangappa, Muthu K. Shanmugam, Srishti Mishra, Arunachalam Chinnathambi, Sulaiman Ali Alharbi, Atanu Bhattacharjee, Gautam Sethi, Alan Prem Kumar, Basappa, Kanchugarakoppal S. Rangappa

**Affiliations:** ^1^Department of Studies in Molecular Biology, University of Mysore, Mysore, India; ^2^Laboratory of Chemical Biology, Department of Chemistry, Bangalore University, Bangalore, India; ^3^Adichunchanagiri Institute for Molecular Medicine, Mandya, India; ^4^Department of Pharmacology, Yong Loo Lin School of Medicine, National University of Singapore, Singapore, Singapore; ^5^Department of Botany and Microbiology, College of Science, King Saud University, Riyadh, Saudi Arabia; ^6^Department of Biotechnology and Bioinformatics, North Eastern Hill University, Shillong, India; ^7^Cancer Science Institute of Singapore, National University of Singapore, Singapore, Singapore; ^8^Department of Studies in Organic Chemistry, University of Mysore, Mysore, India; ^9^Department of Studies in Chemistry, University of Mysore, Mysore, India

**Keywords:** oxadiazoles, NF-κB, hepatocellular carcinoma, apoptosis, anticancer

## Abstract

Aberrant activation of NF-κB is linked with the progression of human malignancies including hepatocellular carcinoma (HCC), and blockade of NF-κB signaling could be a potential target in the treatment of several cancers. Therefore, designing of novel small molecule inhibitors that target NF-κB activation is of prime importance in the treatment of several cancers. In the present work, we report the synthesis of series of 1,3,4-oxadiazoles, investigated their anticancer potential against HCC cells, and identified 2-(3-chlorobenzo[b]thiophen-2-yl)-5-(3-methoxyphenyl)-1,3,4-oxadiazole (CMO) as the lead compound. Further, we examined the effect of CMO on cell cycle distribution (flow cytometry), apoptosis (annexin V-propidium iodide-FITC staining), and phosphorylation of NF-κB signaling pathway proteins (IκB and p65) in HCC cells. We found that CMO induced antiproliferative effect in dose- and time-dependent manner. Also, CMO significantly increased the percentage of sub-G1 cell population and induced apoptosis. Furthermore, CMO found to decrease the phosphorylation of IκB (Ser 32) in the cytoplasmic extract and p65 (Ser 536) in the nuclear extract of HCC cells. It also abrogated the DNA binding ability and transcriptional activity of NF-κB. CMO induced the cleavage of PARP and caspase-3 in a time-dependent manner. In addition, transfection with p65 small interfering RNA blocks CMO-induced caspase-3/7 activation. Molecular docking analysis revealed that CMO interacts with the hydrophobic region of p65 protein. Thus, we are reporting CMO as an inhibitor of NF-κB signaling pathway.

## Introduction

NF-κB is the one of the most widely studied inflammatory mediators associated with several disease conditions including cancer (1, 2). Initially, NF-κB was identified as transcription factor that is essential for the expression of B-cell specific genes and, later, it was demonstrated to present ubiquitously in mammalian cells (3, 4). NF-κB is an inducible transcription factor present in cytoplasm that gets activated in response to inflammation, DNA damage, stress, and viral attack (5). In mammals, RelA, RelB, c-Rel, p50/p105 (NF-κB1), and p52/p100 (NF-κB2) are NF-κB proteins identified so far (6). During the stimuli, these proteins undergo homo/hetero-dimerization, translocate to nucleus, binds to specific DNA elements, and regulate the expression of more than 500 genes that codes for acute phase proteins (Pentraxin-3, Hepcidin, UPA), stress response proteins (COX, LOX, HSP90), apoptosis regulators (Bcl-2, Bcl-xL, Bax), cell adhesion molecules (CD44, ICAM-1, Fibronectin, P-selectin), cytokines (TNF-α, lymphotoxin-a/b, RANTES), growth factors (G-CSF, SCF, Activin-A), and cell surface receptors (ABCA1, CD23, RAGE) (7, 8). In mammals, NF-κB target genes modulate cell proliferation, apoptosis and survival (9, 10). Baltimore et al. demonstrated the role of NF-κB proteins in cell survival by generating the RelA-deficient mice (11). Their experimental results suggested that RelA disruption leads to embryonic lethality at 15–16 days of gestation, with an extensive degeneration of the liver (11). On the other hand, several cancer-causing mutations have been identified in the genes that signal for the activation of NF-κB pathway. Cancer-causing mutations are most likely to contribute for the persistent activation of NF-κB in turn deregulating of expression of NF-κB target genes, which drive the cells to oppose apoptosis (12). Evidently, aberrant activation of NF-κB is reported in several cancers including solid and liquid tumors (13–15). Moreover, constitutive activation of NF-κB has been observed in hepatocellular carcinoma (HCC) tumor tissues suggesting its critical role in the tumorigenesis (16–18). Therefore, targeting activation NF-κB signaling pathway remains as an attractive therapeutic strategy in the fight against cancer.

Several 1,3,4-oxadiazoles have been reported to possess good anticancer potential against various types of cancer cells (19–21). Some of the reports also suggested that oxadiazoles possibly target NF-κB signaling pathway to induce their anticancer activity (22). Specifically, 3-methyl-1-((5-(5-methyl-1,3,4-oxadiazol-2-yl)-2-(thiophen-2-yl)pyrimidin-4-yl)amino)-1H-pyrrole-2,5-dione was reported to possess the IC_50_ value of 0.3 µM for AP-1 and NF-κB mediated transcriptional activation in Jurkat T-cells (22). Although anticancer activity of oxadiazoles is well-documented, the comprehensive study on their putative targets and mechanisms of action has not been reported so far. In continuation of our efforts to explore the anticancer potential of heterocycles (23–26), herein, we report the synthesis of series of novel 1,3,4-oxadiazoles and comprehensively demonstrated their mechanism of anticancer activity *in vitro* against panel of HCC cell lines.

## Materials and Methods

All chemicals used were of analytical grade and purchased from Sigma Aldrich, and SRL, Mumbai (India). ^1^H NMR spectra were recorded on a Agilent (400 MHz) spectrometer in CDCl_3_ solvent, using TMS as an internal standard, ^13^C NMR spectra were recorded on a Agilent (100 MHz) spectrometer. Mass spectra were determined on PE Sciex API3000 ESI-MS, elemental analyses were carried out using an Elemental Vario Cube CHNS rapid analyzer. Progress of the reaction was monitored by TLC pre-coated silica gel plates.

HepG2 cell line was initially purchased from ATCC. The cells were cultured in DMEM medium containing 10% fetal bovine serum, 1 mM l-glutamine, 1 mM sodium pyruvate, antibiotic, and antimycotic agent. GAPDH, lamin B, and p65 antibodies were obtained from Santa Cruz Biotechnology (Santa Cruz, CA, USA). Antibodies against phospho-IκBα (Ser 32), IκBα, phospho-p65 (Ser 536) was purchased from Cell Signaling Technology (Beverly, MA, USA). Nuclear extraction and NF-κB DNA binding kits were purchased from Active motif (USA). Blocking buffer was purchased from Nacalai Tesque (Kyoto, Japan). Chemiluminescence kit was purchased from Advansta (CA, USA). The small interfering RNA (siRNA) for NF-κB and scrambled control was obtained from Santa Cruz Biotechnology. Caspase-Glo 3/7 assay kit and luciferase substrate was purchased from promega (WI, USA).

### Chemistry

#### General Procedure for the Preparation of Acid Hydrazide (3a–c)

The appropriate aromatic acids (0.01 mol) were dissolved in absolute ethanol (10 ml) followed by the addition of hydrazine hydrate (0.02 mol) and 2–3 drops of conc. sulfuric acid. The reaction mixture was refluxed for 7 h. The completion of reaction was monitored by thin layer chromatography, and the resulting solid obtained was filtered, dried, and crystallized (3a-c).

#### General Procedure for the Synthesis of 1,3,4-Oxadiazole (5a–l)

The aromatic acid hydrazide (0.01 mol) and an appropriate aromatic acid (0.01 mol) were refluxed in phosphorous oxychloride (5 ml) for 8 h, and the reaction mixture was cooled to room temperature. Excess POCl_3_ was removed through high vacuum, the residue was quenched with ice and made alkaline with potassium carbonate solution. The precipitate was filtered, dried, and crystallized from ethanol. The completion of reaction was monitored by thin layer chromatography. The representative spectra of some of the new compounds are provided as Supplemental Information.

##### 4-(5-(3-Chlorobenzyl)-1,3,4-Oxadiazol-2-yl)-*N*,*N*-Dimethylaniline (5a)

Yellow solid; mp 110–112°C; 71% yield; ^1^H NMR (400 MHz, CDCl_3_): δ = 7.84–7.81 (d, 2H, Ar-H), 7.34 (s, 1H, Ar-H), 7.22–7.25 (m, 3H, Ar-H), 6.70–6.68 (d, 2H, Ar-H), 4.19 (s, 2H, –CH_2_), 3.02 (s, 6H, 2CH_3_); ^13^C (100 MHz CDCl_3_): δ = 152.37, 136.19, 134.64, 130.05, 128.96, 128.20, 127.65, 126.96, 111.54, 40.00, and 31.49 ppm; exact mass: 313.0, ESI mass [M + 1] 314.1; Anal. Calcd for C_17_H_16_ClN_3_O: C, 65.07; H, 5.14; N, 13.39; found: C, 65.23; H, 5.11; N, 13.11.

##### 4-(5-Benzyl-1,3,4-Oxadiazol-2-yl)-*N*,*N*-Dimethylaniline (5b)

Yellow solid; mp 154–156°C; 79% yield; ^1^H NMR (400 MHz, CDCl_3_): δ = 7.84–7.82 (d, 2H, Ar-H), 7.25–7.34 (m, 5H, Ar-H), 6.72–6.69 (d, 2H, Ar-H), 4.23 (s, 2H, -CH_2_), 3.04 (s, 6H, 2CH_3_); ^13^C (100 MHz CDCl_3_): δ = 134.34, 128.80, 128.77, 128.18, 127.33, 111.68, 40.10, and 31.88 ppm; exact mass: 279.1, ESI mass [M + 1] 280.2; Anal. Calcd for C_17_H_17_N_3_O: C, 73.10; H, 6.13; N, 15.04; found: C, 73.34, H, 5.88, N, 15.24.

##### 4-(5-(4-Bromobenzyl)-1,3,4-Oxadiazol-2-yl)-*N*,*N*-Dimethylaniline (5c)

Yellow solid; mp 107–109°C; 82% yield; ^1^H NMR (400 MHz, CDCl_3_): δ = 7.82–7.80 (d, 2H, Ar-H), 7.47–7.42 (t, 2H, Ar-H), 7.25–7.21 (t, 2H, Ar-H), 6.70–6.68 (d, 2H Ar-H), 4.18 (s, 2H, -CH_2_), 3.02 (s, 6H, 2CH_3_); exact mass: 357.1, ESI mass [M + 2] 359.6; Anal. Calcd for C_17_H_16_BrN_3_O: C, 57.00; H, 4.50; N, 11.73; found: C, 56.98; H, 4.32; N, 11.81.

##### 4-(5-(3-Chlorobenzo[b]thiophen-2-yl)-1,3,4-Oxadiazol-2-yl)-*N*,*N*-Dimethylaniline (5d)

Yellow solid; mp 120–123°C; 86% yield; ^1^H NMR (400 MHz, CDCl_3_): δ = 8.04–8.02 (d, 1H), 7.96–7.82 (m, 3H), 7.52–7.49 (m, 2H), 6.91–6.88 (d, 2H), 3.08–3.05 (t, 6H); exact mass: 355.1, ESI mass: [M + 1] 356.3, Anal. Calcd for C_18_H_14_ClN_3_OS: C, 60.76; H, 3.97; N, 11.81; found: C, 60.58; H, 3.88, N, 11.52.

##### 3-(5-(4-(Dimethylamino)phenyl)-1,3,4-Oxadiazol-2-yl)-*N*-(2-Methyl-3-(Trifluoromethyl) phenyl)pyridin-2-amine (5e)

Yellow solid; mp 170–173°C; 78% yield; ^1^H NMR (400 MHz, CDCl_3_): δ = 8.31–8.21 (m, 3H, Ar-H), 7.99–7.98 (d, 2H, Ar-H), 7.45–7.25 (m, 2H, Ar-H), 6.88–6.85 (m, 1H, Ar-H), 6.78–6.76 (d, 2H, Ar-H), 3.07–3.05 (d, 6H, 2CH_3_), 2.53 (s, 3H, -CH_3_); ^13^C (100 MHz CDCl_3_): δ = 164.57, 161.92, 153.26, 152.62, 150.61, 139.56, 136.00, 128.48, 127.16, 125.69, 121.52, 121.47, 113.70, 111.68, 110.18, 103.53, 40.04, 14.28; exact mass: 439.2 [M + 1] 440.3; Anal. Calcd for C_23_H_20_F_3_N_5_O: C, 62.86; H, 4.59; N, 15.94; found: C, 62.91, H, 4.42; N, 15.63.

##### 2-(4-Bromobenzyl)-5-(6-Chloropyridin-3-yl)-1,3,4-Oxadiazole (5f)

Brown solid; mp 110–112°C; 83% yield; ^1^H NMR (400 MHz, CDCl_3_): δ = 8.97–8.96 (d, 1H), 8.26–8.23 (m, 1H), 7.50–7.43 (m, 3H), 7.25–7.21 (t, 2H), 4.24 (s, 2H); exact mass: 348.9, ESI Mass: [M + 2] 351.3; Anal. Calcd for C_14_H_9_BrClN_3_O: C, 47.96; H, 2.59; N, 11.99; found: C, 47.81, H, 2.49, N, 12.13.

##### 2-(4-Bromobenzyl)-5-(Pyridin-3-yl)-1,3,4-Oxadiazole (5g)

Brown solid; mp 133–135°C; 71% yield; ^1^H NMR (400 MHz, CDCl_3_): δ = 9.97 (s, 1H), 8.32–8.31 (d, 1H), 8.23–8.21 (t, 1H), 7.99–7.97 (d, 2H), 7.32–7.25 (t, 1H), 6.78–6.76 (d, 2H), 3.08 (s, 2H); exact mass: 315.0, ESI mass: [M + 2] 317.3, Anal. Calcd for C_14_H_10_BrN_3_O: C, 53.19; H, 3.19; N, 13.29; found: C, 53.10; H, 2.99; N, 13.42.

##### 2-Benzyl-5-(4-Bromobenzyl)-1,3,4-Oxadiazole (5h)

Brown solid; 123–124°C; 86% yield; ^1^H NMR (400 MHz, CDCl_3_): δ = 7.83–7.81 (d, 2H), 7.47–7.43 (t, 2H), 7.25–7.21 (m, 2H), 6.72–6.70 (d, 2H), 4.02–4.05 (m, 4H); exact mass: 328.0, ESI mass: [M + 2] 330.5; Anal. Calcd for C_16_H_13_BrN_2_O: C, 58.38; H, 3.98; N, 8.51; found: C, 58.19, H, 3.88; N, 8.67.

##### 2-(3-Methoxyphenyl)-5-(4-(Trifluoromethyl)phenyl)-1,3,4-Oxadiazole (5i)

White solid; mp 100–102°C; 77% yield; ^1^H NMR (400 MHz, CDCl_3_): δ = 7.98–7.43 (m, 7H), 7.12–7.09 (m, 1H), 3.19 (s, 3H); ^13^C (100 MHz CDCl_3_): δ = 160.02, 138.15, 130.29, 127.79, 125.75, 124.56, 123.28, 122.73, 119.59, 119.33, 118.15, 111.91, 55.54; exact mass: 320.1 ESI mass: [M + 1] 321.3; Anal. Calcd for C_16_H_11_F_3_N_2_O_2_: C, 60.00; H, 3.46; N, 8.75; found: C, 60.17, H, 3.41, N, 8.69.

##### 2-(3-Chlorobenzo[b]thiophen-2-yl)-5-(3-Methoxyphenyl)-1,3,4-Oxadiazole (CMO, 5j)

White solid; mp 112–114°C; 72% yield; ^1^H NMR (400 MHz, CDCl_3_): δ = 7.44–8.00 (m, 7H), 7.12–7.10 (m, 1H), 3.91 (s, 3H); ^13^C (100 MHz CDCl_3_): δ = 160.33, 158.67, 155.32, 125.53, 125.43, 122.62, 122.48, 121.47, 121.38, 121.34, 119.91, 114.66, 114.58, 113.66, 113.39, 107.12, and 50.79 ppm; exact mass: 342.0, ESI mass: [M + 1] 343.1; Anal. Calcd for C_17_H_11_ClN_2_O_2_S: C, 59.56; H, 3.23; N, 8.17; found: C, 59.42; H, 3.32, N, 8.23.

### Pharmacology

#### MTT Assay

The antiproliferative effect of newly synthesized compounds against HCC cells was determined by the MTT dye uptake method as described previously (27, 28). Briefly, HCC cells (4 × 10^3^ cells/well) were incubated in triplicate in a 96-well plate, in the presence of different concentrations of compounds at a volume of 0.2 ml, for different time intervals at 37°C. Thereafter, a 20 µl MTT solution (5 mg/ml in PBS) was added to each well. After a 2 h incubation at 37°C, 0.1 ml lysis buffer (20% SDS, 50% dimethylformamide) was added; incubation was performed for 1 h at 37°C, and the optical density (OD) at 570 nm was measured by Tecan plate reader. 0.01% DMSO was used as the negative control and 0.01% MTT was used as a control agent.

#### Flow Cytometric Analysis

Flow cytometric analysis was performed to evaluate apoptosis inducing effect of CMO in HepG2 and HCCLM3 cells as described earlier (29, 30). Briefly, HCC cells (5 × 10^5^) were plated in petri dish and, 24 h later, the cells were exposed to compound CMO (50 µM) for 0, 24, 48, and 72 h. Thereafter, cells were washed, fixed with 70% ethanol, and incubated for 30 min at 37°C with 0.1% RNase A in PBS. Cells were washed again, resuspended, and stained with PBS containing 25 µg/ml propidium iodide (PI) for 15 min at room temperature. The cell cycle distribution across the various phases was analyzed using flow cytometer.

#### Annexin V/PI Apoptosis Assay

Phosphatidylserine exposure and cell death were assessed by FACS analysis using Annexin-V-PI-stained cells as described previously (31). Briefly, 1 × 10^5^ HepG2 cells/well (190 μl/well) were seeded in 96-well plates and incubated with CMO (50 µM) for indicated time points (24, 48, and 72 h), and DMSO treated samples were used as control. Cells were then washed with Annexin V binding buffer (10 mM HEPES/NaOH, pH 7.4, 140 mM NaCl, 2.5 mM CaCl_2_), stained with Annexin V FITC for 30 min at room temperature in the dark, then washed again, and re-suspended in Annexin V binding buffer containing PI. Samples were analyzed immediately.

#### Immunoblotting Assay

For detection of phophoproteins, control and CMO treated cells, cytoplasmic and nuclear extract were prepared for according to manufacturer’s instructions. Lysates were then spun at 14,000 rpm for 10 min to remove insoluble material and resolved on a 10% SDS gel. After electrophoresis, the proteins were electrotransferred to a nitrocellulose membrane, blocked with blocking buffer, and probed with primary antibodies overnight at 4°C. The blot was washed, exposed to HRP-conjugated secondary antibodies for 1 h, and finally examined by chemiluminescence and imaged using ChemiDoc™ imaging system (BioRad, USA).

#### NF-κB DNA Binding Assays

To determine NF-κB activation, we performed DNA-binding assay using TransAM NF-κB kit according to the manufacturer’s instructions and as previously described (32). Briefly, 50 µg of nuclear proteins were added into 96-well plate coated with an unlabeled oligonucleotide containing the consensus binding site for NF-κB (5′-GGGACTTTCC-3′) and incubated for 4, 8, and 12 h. The wells were washed and incubated with antibodies against NF-κB p65 subunit. An HRP conjugated secondary antibody was then applied to detect the bound primary antibody and provided the basis for colorimetric quantification. The enzymatic product was measured at 450 nm by microplate reader (Tecan Systems).

#### NF-κB Luciferase Reporter Assay

The effect of CMO on constitutive a NF-κB-dependent reporter gene transcription in HepG2 and HCCLM3 cells was determined as previously described (32). NF-κB responsive elements linked to a luciferase reporter gene were transfected with wild-type or dominant-negative IκB. The transfected cells were then treated with CMO for 4, 8, and 12 h. Luciferase activity was measured with a Tecan (Durham, NC, USA) plate reader and normalized to β-galactosidase activity. All luciferase experiments were done in triplicate.

#### p65 siRNA Transfection

HepG2 cells were plated in 6-well plates and allowed to adhere for 24 h. On the day of transfection, lipofectamine was added to control or p65 siRNA in a final volume of 1 ml of culture medium. After 48 h of incubation following transfection, HepG2 cells (1 × 10^4^ cells/well) in 6-well plate were treated with CMO for 24 h. Equal volume of Caspase-Glo^®^ 3/7 reagent was then added to the wells to provide the 1:1 ratio of reagent volume to sample volume. After incubation for 15–30 min at room temperature, the luminescence was measured by Tecan microplate reader and activation of caspase-3/7 was analyzed.

#### Caspase-Glo^®^ 3/7 Luminescent Assay

Caspase activity was measured using Caspase-Glo^®^ 3/7 assay kit (Promega) according to the manufacturer’s instructions.

#### *In Silico* Interaction Analysis

Discovery Studio 2.5 software from Accelrys was used for docking and visualization of the results as described earlier (33, 34). Initially, we retrieved the crystal structure of NF-κB complex (PDB: 1IKN) (35), cleaned, minimized the energy, and identified the spatial region of p65. All the energy calculations were performed using CHARMM force field. The three-dimensional structures of all oxadiazoles were prepared and docked toward the p65 using LIGANDFIT protocol of Discovery Studio. The binding pose of ligands was evaluated using the interaction score function in the Ligand Fit module of Discovery Studio as reported previously (36).

## Results and Discussion

### Chemistry

**Scheme 1:**
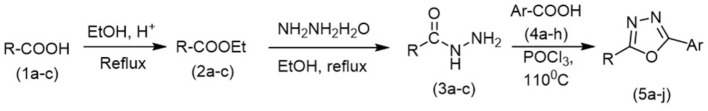
Schematic representation for the synthesis of 1,3,4-oxadiazoles.

Initially, carboxylic acid (1a-c) was converted to their corresponding ester (2a-c) followed by refluxing with hydrazine hydrate in ethanol, which resulted in the formation of acid hydrazides (3a-c). Thereafter, 1,3,4-oxadiazoles (5a-j) were synthesized by refluxing equimolar mixture of acid hydrazide (3a-c), with different aromatic carboxylic acid (4a–h) in phosphorous oxychloride (5 ml) for 7 h (Scheme 1). The structures of all the target compounds (Table 1) were characterized by elemental analysis LCMS, ^1^H NMR and ^13^C NMR spectrometry.

**Table 1:** Library of newly synthesized 1,3,4-oxadiazoles.

Entry	Acid hydrazide	Carboxylic acid	Oxadiazole
1	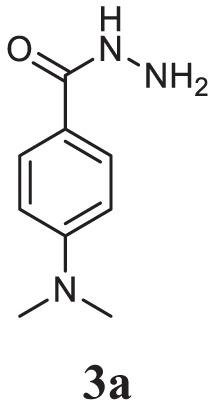	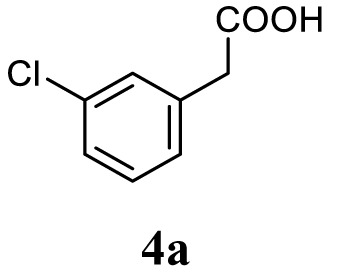	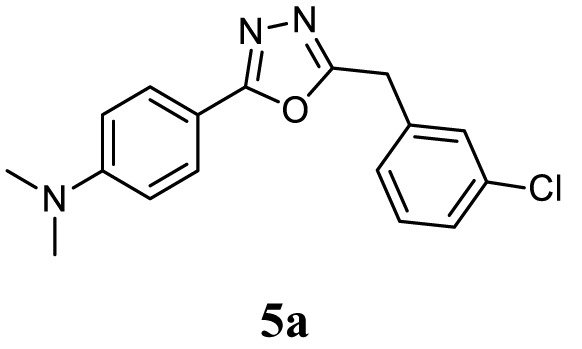
2		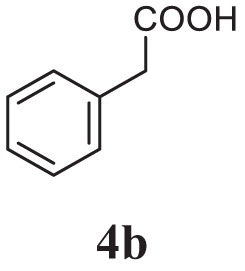	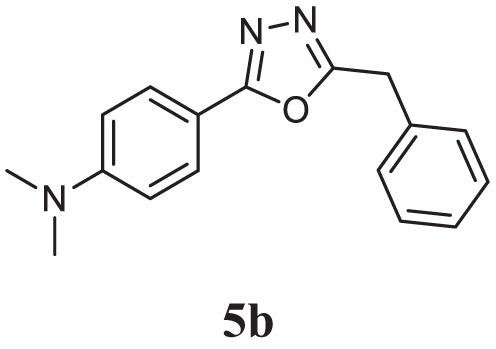
3		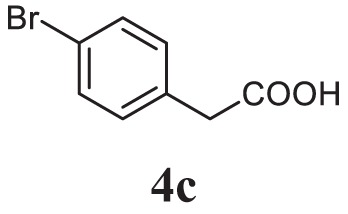	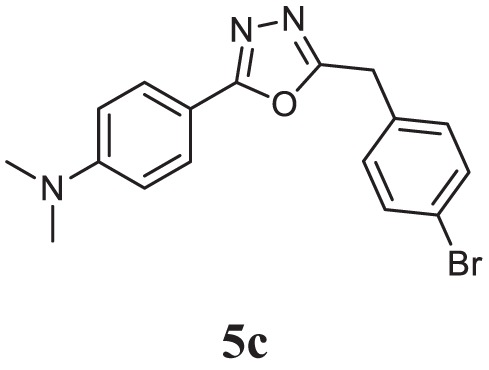
4		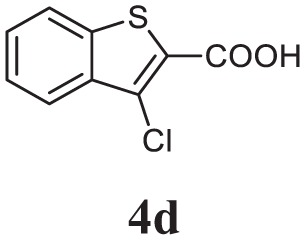	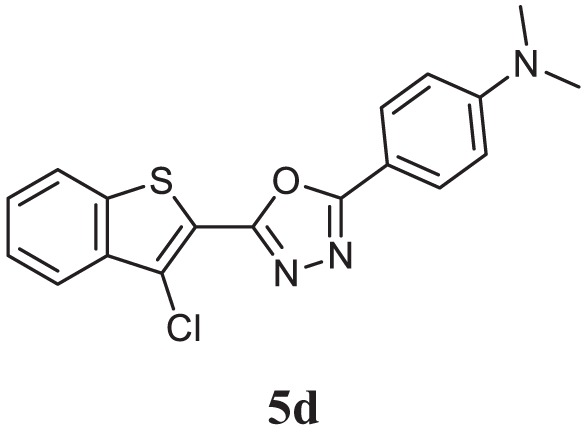
5		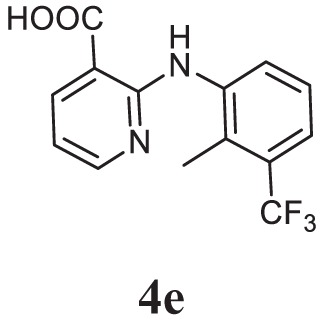	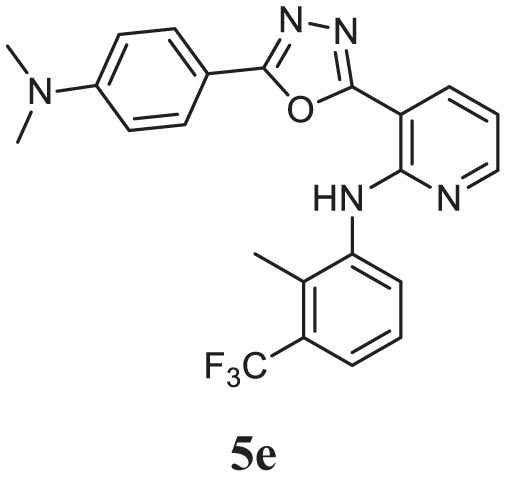
6	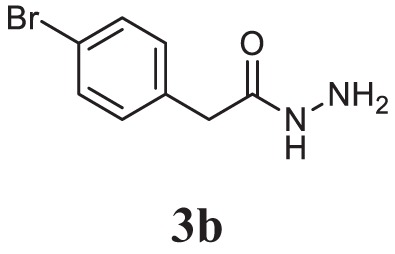	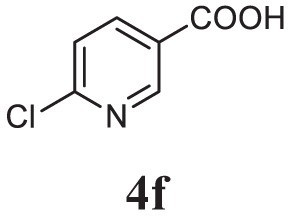	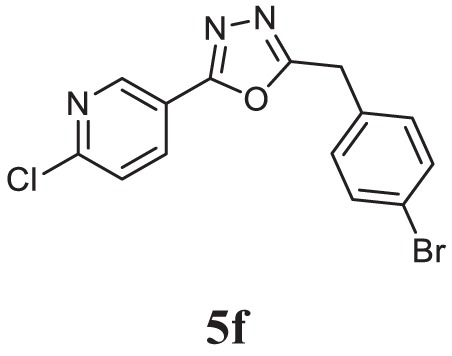
7		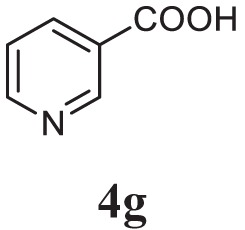	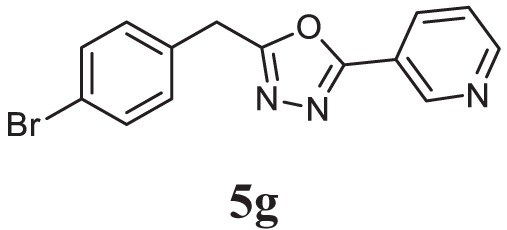
8		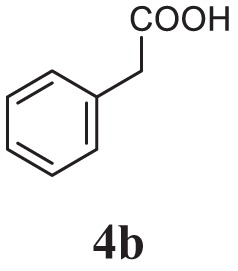	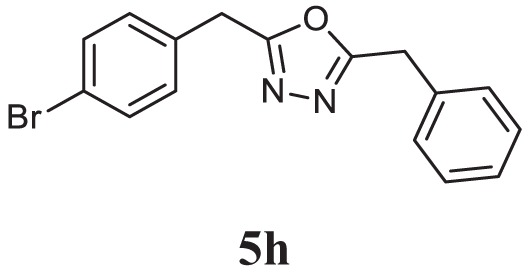
9	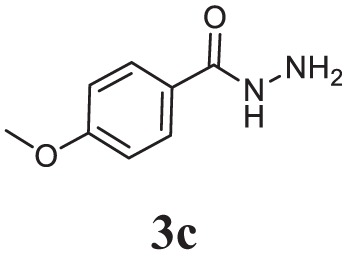	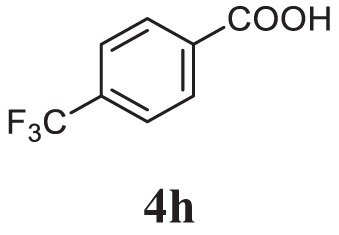	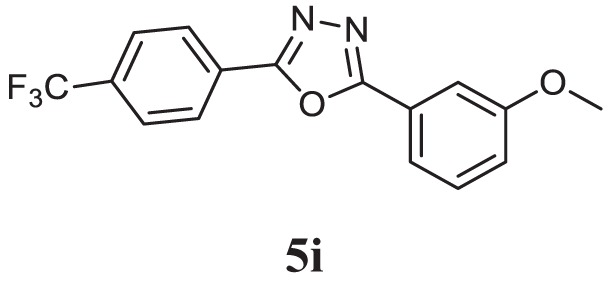
10		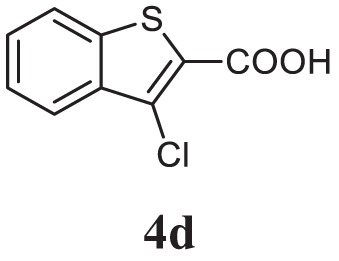	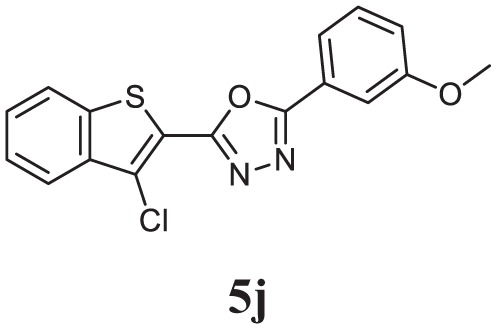

### Pharmacology

#### 1,3,4-Oxadiazoles Mitigate the Proliferation of HCC Cells in Time- and Dose-Dependent Manner

**Figure 1:**
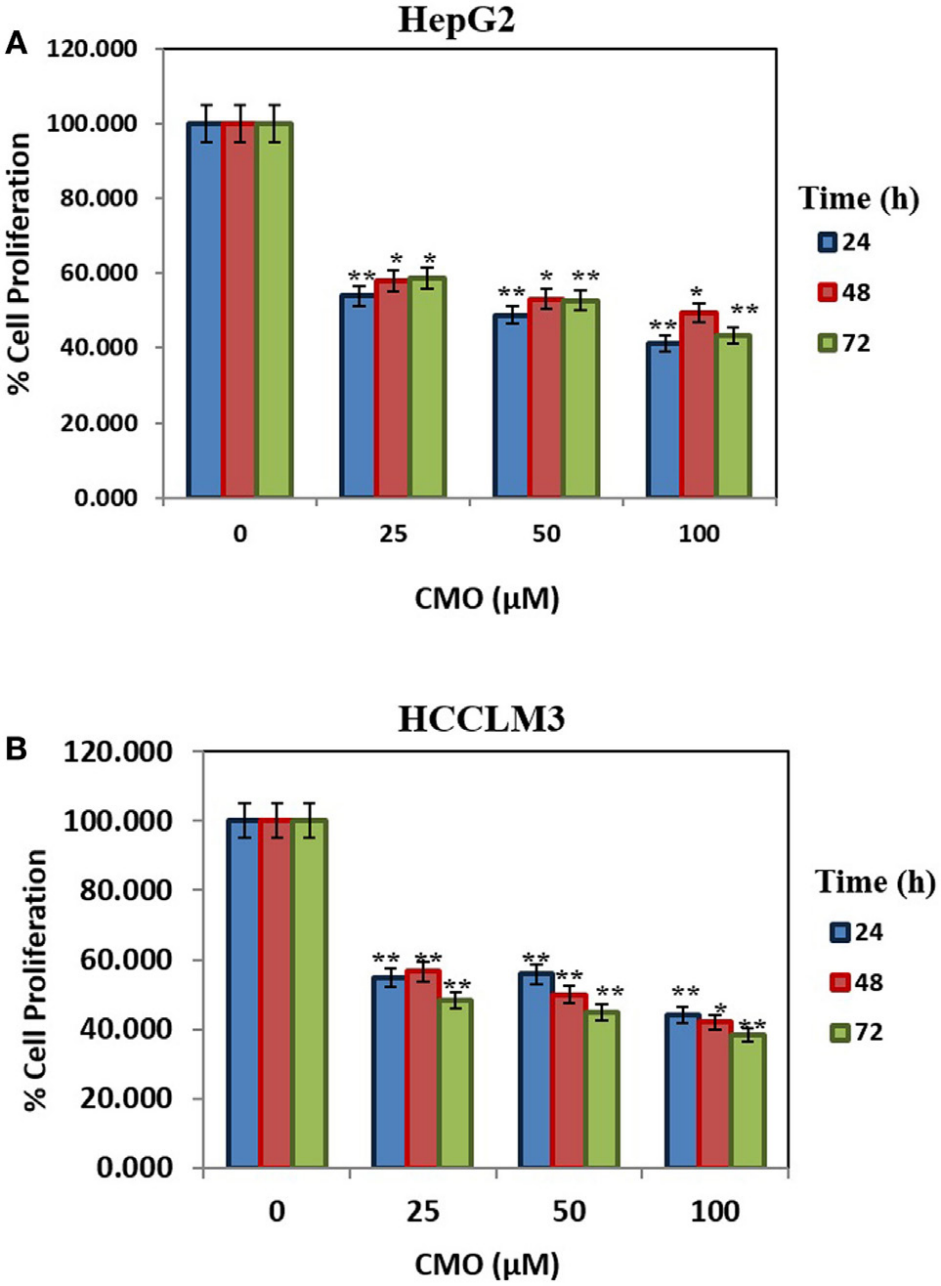
**(A,B)** CMO induces antiproliferative effect in time- and dose-dependent manner. HepG2 and HCCLM3 cells were plated in triplicate, treated with indicated concentrations of CMO, and then subjected to MTT assay after 24, 48, and 72 h to analyze proliferation of cells. The data are expressed as mean ± SD, compared with the untreated control (*p < 0.05, **p < 0.01).

Initially, we prepared the library of 10 novel 1,3,4-oxadiazoles and screened the newly synthesized compounds for their antiproliferative potential against HepG2 and HCCLM3 cells by MTT assay (37). Among the screened compounds, CMO was identified as the most potent antiproliferative agent with an IC_50_ of 27.5 µM against HCCLM3 cells. Our results presented that CMO possess relatively higher antiproliferative efficacy against HCCLM3 than HepG2 cells. We next treated HCCLM3 and HepG2 cells with different concentrations of CMO for different time intervals. We observed a significant reduction in proliferation of cells in a dose- and time-dependent manner (Figures 1A,B).

#### CMO Causes Increased Accumulation of HCC Cells in subG1 Phase

**Figure 2:**
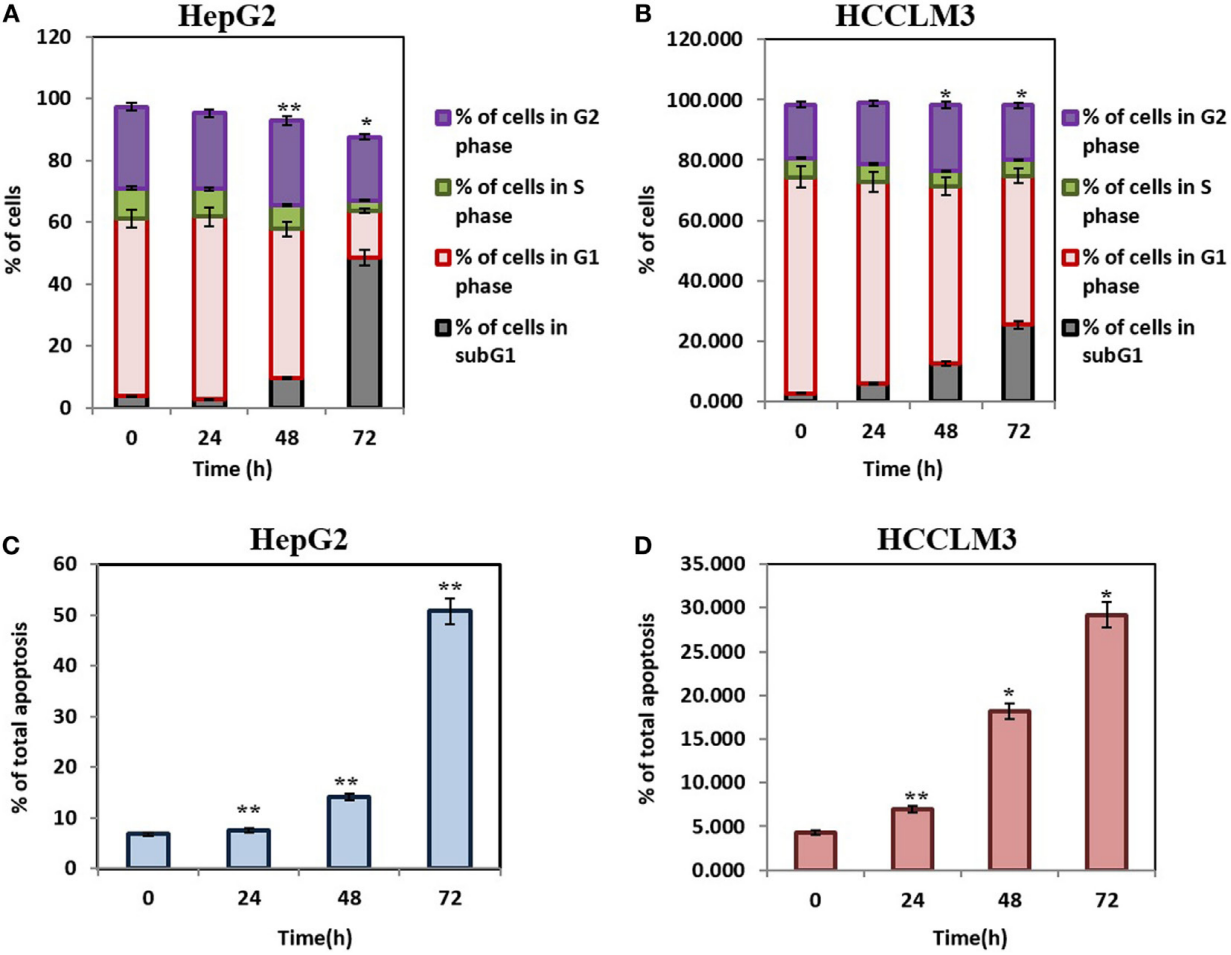
**(A,B)** CMO induces accumulation of hepatocellular carcinoma (HCC) cells in SubG1 phase. HepG2 and HCCLM3 cells were treated with 50 µM of CMO for 24, 48, and 72 h, after which, the cells were washed, fixed, stained with propidium iodide, and subjected to flow cytometric analysis. **(C,D)** CMO induces substantial apoptosis in HCC cells. HepG2 and HCCLM3 cells were exposed to 50 µM CMO at indicated times, after which, cells were harvested and stained with Annexin V and propidium iodide. The percentage of early and late apoptosis in the HCC cells treated with 50 µM CMO was examined using flow cytometry. The bar graph shows total percentage of apoptotic cells of HepG2 and HCCLM3 upon treatment with 50 µM of CMO at the indicated time points. The data are expressed as mean ± SD, compared with the untreated control (*p < 0.05, **p < 0.01).

Caspase-activated DNase-mediated fragmentation of the genomic DNA is remarkable event in the cells committed to undergo apoptosis, which results in the formation of cells with lesser DNA content (38). These cells are termed as hypodiploid cells and can be detected as subG1 cell population in flow cytometric analysis (39). In order to investigate the effect of CMO on distribution of cell cycle, HepG2 and HCCLM3 cells were treated with CMO at 50 µM for different time points up to 72 h and analyzed cell cycle distribution after PI staining. The results demonstrated that CMO significantly increased the subG1 cell population of HCCLM3 to 6, 12.5, and 25.3% at 24, 48, and 72 h, respectively. The cells in subG1 phase of HepG2 were found to be 2.8, 9.66, and 48.6% at 24, 48, and 72 h, respectively (Figures 2A,B).

#### CMO Induces Apoptosis in HCC Cells

The exposure of phosphatidylserine in the outer leaflet of plasma membrane is the most common biochemical change that is observed in the apoptotic cells, which can be detected using annexin V-FITC-PI staining (40). To ensure that CMO induces antiproliferative effect *via* apoptosis, we next examined the effect of CMO externalization of phosphatidylserine in HCCLM3 cells. Interestingly, the treatment with CMO significantly increased the percentage of early (annexin V-positive and PI-negative cells) and late (annexin V-positive and PI-positive cells) apoptotic cells compared with DMSO-treated cells (Figures 2C,D). These results demonstrate that CMO induce apoptosis in HCC cells.

#### CMO Inhibits the Phosphorylation of IκB and Depletes the Nuclear Pool of p65 in HCC Cells

**Figure 3:**
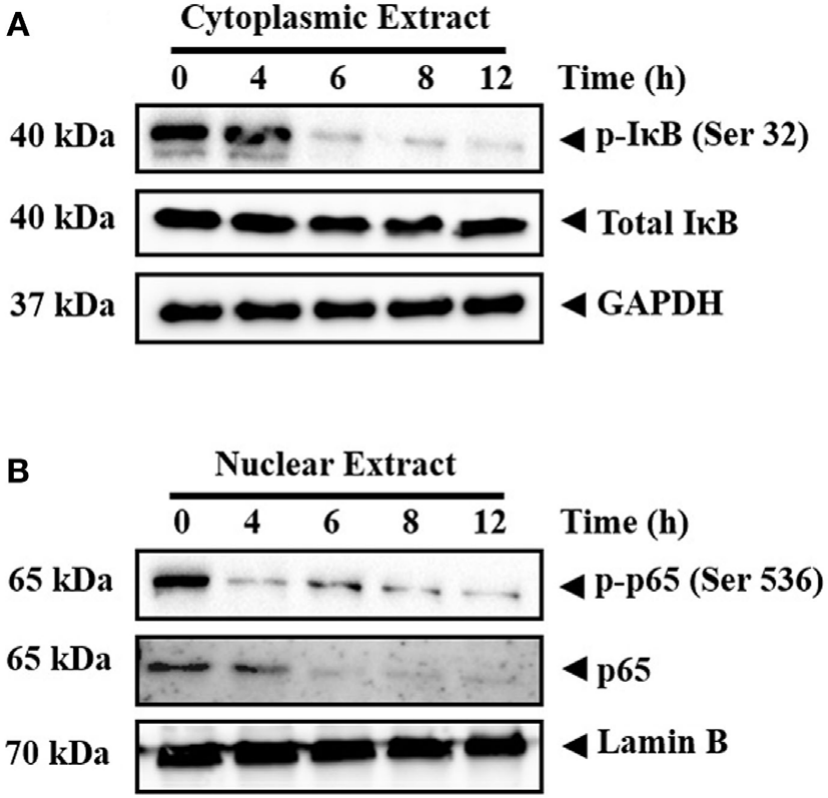
**(A,B)** CMO inhibits the phosphorylation of IκB and depletes the nuclear pool of p65 in hepatocellular carcinoma cells. HepG2 cells were treated with 25 µM CMO for indicated time point, after which, the cytoplasmic and nuclear extracts were prepared, and protein was resolved on SDS-PAGE gel, electrotransferred onto nitrocellulose membranes, and probed for phospho-IκB, IκB, phospho-p65, p65, GAPDH, and Lamin B antibodies.

Inactive NF-κB in association with inhibitory kappa B (IκB) is present in the cytoplasm (41). Phosphorylation and proteolytic degradation of IκB is essential for posttranslational activation of NF-κB and, upon activation, NF-κB translocate to nucleus to induce the expression of target genes (42). In order to investigate the effect of CMO on NF-κB signaling pathway, HepG2 cells were treated with CMO for different time points up to 12 h, prepared the cytoplasmic extract, and analyzed the levels of phospho-IκB. Interestingly, we found the decrease in phosphorylation of IκB (Ser 32) in a time-dependent manner (Figure 3A). At the same time, IκB and GAPDH protein expression remained unchanged.

The phosphorylation of p65 (Ser 536) defines an IκB-independent NF-κB signaling pathway (43). Therefore, we further examined the levels of phospho-p65 in the nuclear extract of cells treated with CMO at different time points up to 12 h. The results clearly demonstrated the decline in phospho-p65 and p65 in a time-dependent manner (Figure 3B). The expression of lamin B was used as input control, which remained unaltered.

#### CMO Abrogated NF-κB DNA Binding and Luciferase Activity in HCC Cells

**Figure 4:**
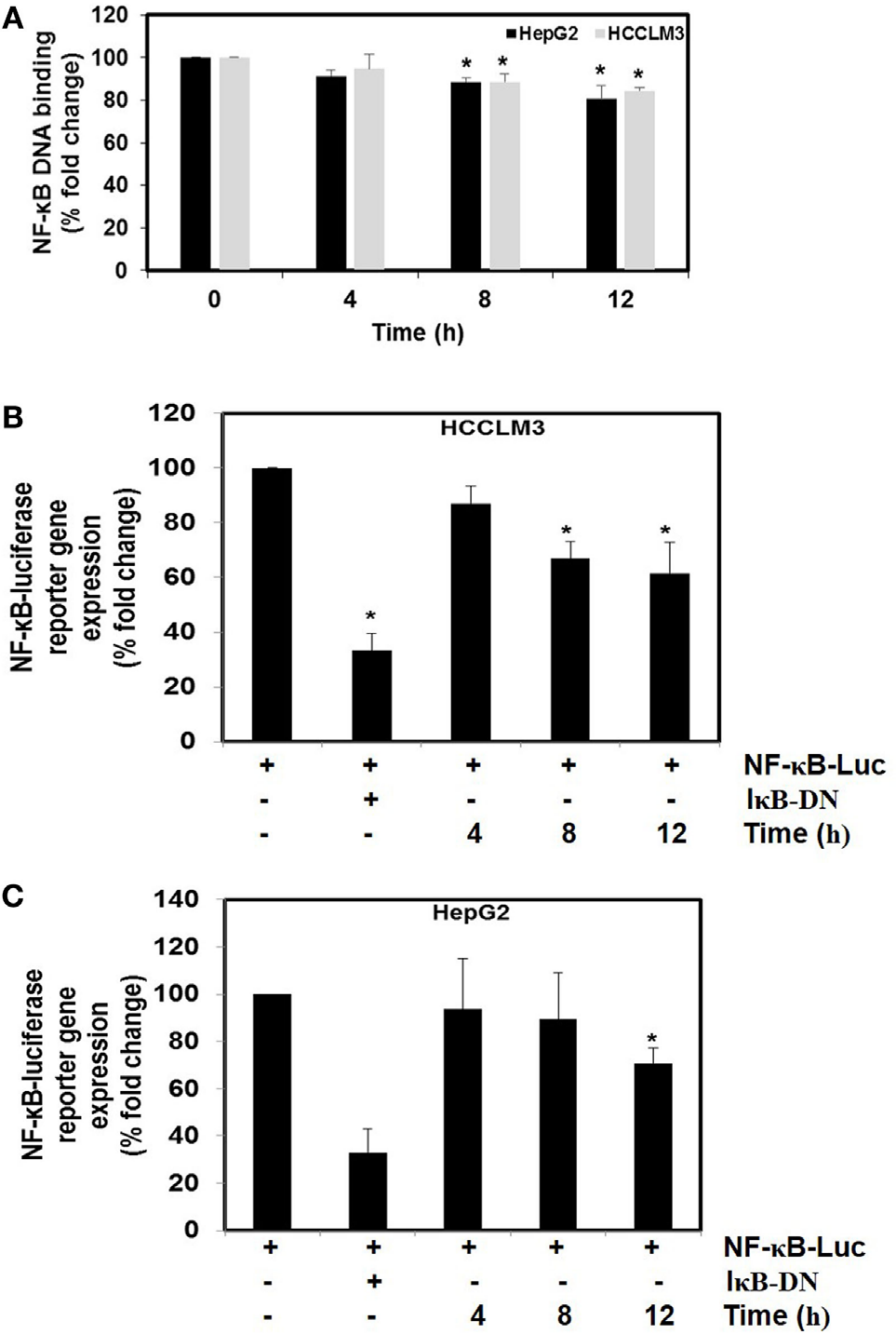
**(A)** The effect of CMO on constitutive NF-κB DNA-binding activity. HepG2 cells (5 × 10^5^/ml) and HCCLM3 cells (5 × 10^5^/ml) were treated with CMO for 4, 8, and 12 h. Nuclear extracts were prepared, 50 µg of the nuclear extract protein was taken for DNA-binding assay as described in Section “Materials and Methods.” **(B,C)** CMO inhibits constitutive activation of reporter gene expression. HepG2 (5 × 10^5^/ml) and HCCLM3 (5 × 10^5^/ml) cells were transfected with NF-κB luciferase and β-galactosidase reporter plasmid using lipofectamine, incubated for 24 h, and then treated with CMO for 4, 8, and 12 h. Cells were lysed in reporter lysis buffer and analyzed for luciferase activity and normalized with β-galactosidase activity. Results are expressed as % fold activity over the activity of vector control. *p < 0.05.

We next investigated the effect of CMO on constitutive NF-κB activity in HCC cells. Cells were preincubated with 25 µM CMO for 4, 8, and 12 h and then nuclear extracts were prepared and tested for NF-κB DNA-binding activity. We noted that treatment with CMO suppressed constitutive NF-κB activity in a time-dependent manner (Figure 4A). To analyze the effect of CMO on constitutive NF-κB-dependent reporter gene expression in HCC cells, transfection was done as described in Section “Materials and Methods.” In the presence of CMO, NF-κB-dependent luciferase expression was significantly reduced in a time-dependent manner with maximum inhibition at 12 h (Figures 4B,C). These results further demonstrate that CMO can abrogate constitutive NF-κB activation in HCC cells.

#### Transfection with p65 siRNA Blocks CMO Induced Caspase-3/7 Activation

**Figure 5:**
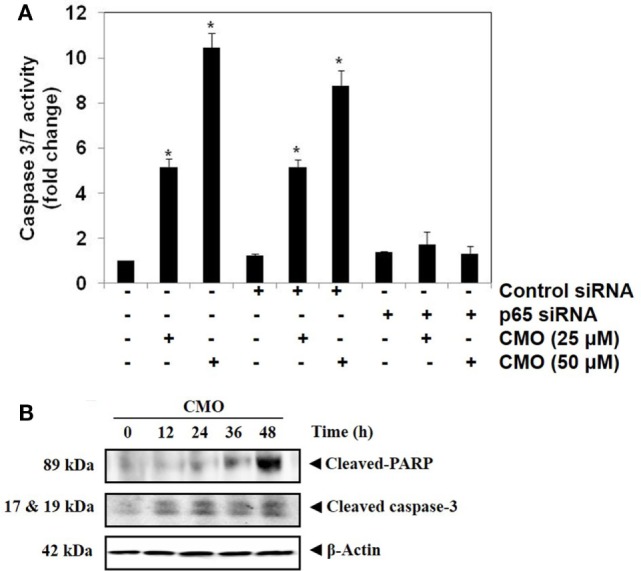
**(A)** Knockdown of p65 by small interfering RNA (siRNA) reduces the apoptotic effect of CMO. HepG2 cells were transfected with either control or p65 specific siRNA (50 nM). After 48 h, the cells were treated with CMO (25 or 50 µM) for 24 h, and the enzymatic activity of caspase-3/7 was determined by Caspase-Glo^®^ 3/7 assay kit. **(B)** CMO increases the cleavage of PARP and Caspase 3 in HCCLM3 cells. HCCLM3 cells were treated with 50 µM CMO for 12, 24, 36, and 48 h, after which, the whole-cell extracts were prepared, and 30 µg of protein was resolved on 12% SDS-PAGE gel, electrotransferred onto nitrocellulose membranes, and probed for cleaved PARP and cleaved caspase 3 antibodies. The data are expressed as mean ± SD, compared with the untreated control (*p < 0.05).

We determined whether the knockdown of p65 using siRNA could significantly block the increase in CMO induced caspase-3/7 activation in HepG2 cells. In cells transfected with control siRNA, CMO treatment significantly increased caspase-3/7 activation, thereby inducing apoptosis (Figure 5A). The results clearly indicate that the observed increase in caspase-3/7 activation was significantly suppressed in the cells transfected with p65 siRNA when compared to control siRNA treated group.

#### CMO Induces the Cleavage of PARP and Caspase-3

During apoptosis, procaspase-3 is cleaved to form active caspase 3 (executioner caspase), which in turn cleaves the full-length PARP (116 kDa) into 85- and 24-kDa fragments. PARP is associated with DNA repair mechanism, and its cleavage drives the cell to apoptosis. We next evaluated whether inhibition of NF-κB by CMO induce PARP and caspase-3 cleavage. We observed the increase in cleaved caspase-3 and PARP demonstrating that CMO induces caspase-3-mediated apoptosis, which is in agreement with the results of previous experiments (Figure 5B).

#### *In Silico* Analysis of Oxadiazoles with p65

**Figure 6:**
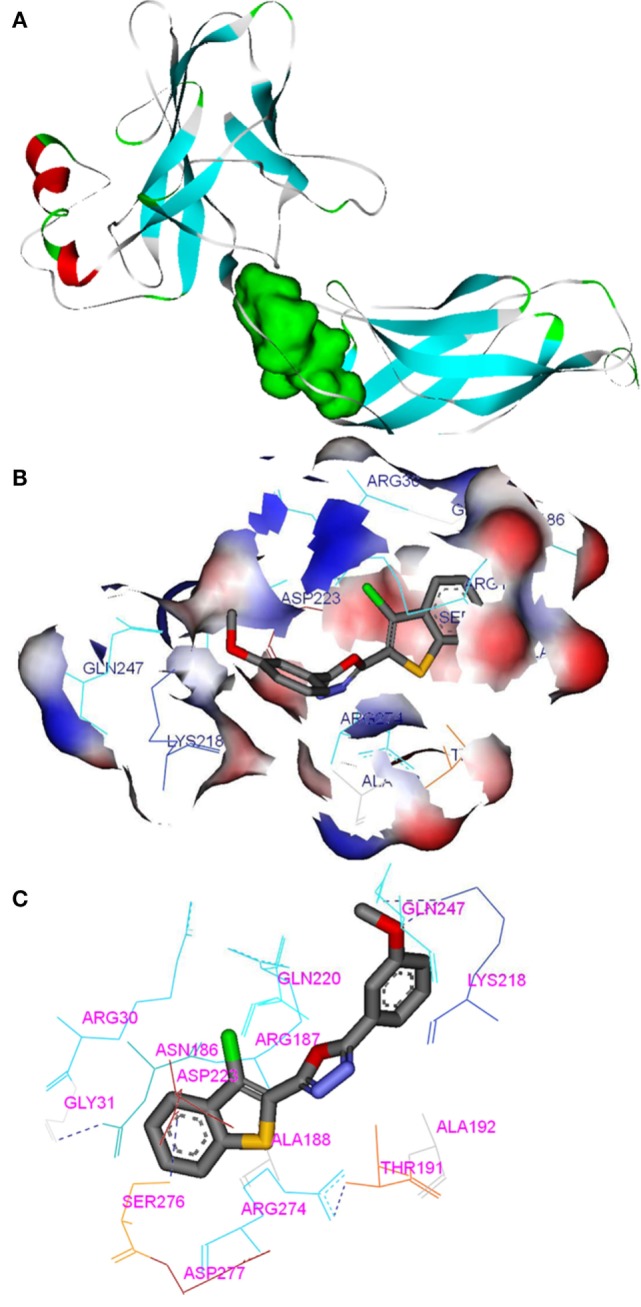
*In silico* molecular interactions between p65 of NF-κB complex and the oxadiazole derivatives: **(A)** hydrophobic region of p65 was shown as green mass; **(B)** surface view of CMO bound p65 with the key amino acids (labeled); **(C)** interaction map and hydrogen bonding (dotted line) pattern of p65 protein with CMO.

Compounds of the type 1,3,4-oxadiazoles are known to target NF-κB in several proinflammatory disease models. It was also demonstrated that alkylating agent such as *N*-ethylmaleimide and oxidizing agent eliminated the DNA binding ability of NF-κB (44). In another study, helenalin (the sesquiterpene lactone) selectively alkylates p65 subunit and inhibits the activation of NF-κB (45). Helenalin is an oxygen containing heterocycle with good NF-κB inhibitory activity. Based on these reports, we predicted that electronegativity of oxygen and nitrogen and electropositivity of other atoms in the oxadiazole ring contributes for inhibiting the activation of NF-κB. To test the hypothesis and in order to understand the interaction of 1,3,4-oxadiazoles toward NF-κB, the crystal structure of NF-κB complex was considered in our study. Accelrys Discovery Studio default tools and settings (version 2.5) were used for the molecular docking procedures. Further, the hydrophobic region near the Cys38 of p65 protein was identified using Accelrys binding site identification tool. Using the LIGANDFIT protocol of the ligand–receptor interaction module of Discovery Studio version 2.5, the oxadiazoles were docked into the hydrophobic region of p65 (Figure 6A), and the docking scores of all the compounds were summarized (Table 2). Docking scores indicated that CMO binds to the p65 with the higher value of (and thus most favorable) score of 61.0 kcal/mol. The interaction pattern revealed that benzothiazole ring of CMO enters the hydrophobic region of p65 and interacts with Asn186, Arg187, Ala188 on one side and Arg30, Gly31, Asp223, Arg274, ser276, Asp277 on the other side. Further, oxadiazole ring of CMO is found to interact with Ala190, Thr191, and Gln220 (Figures 6B,C). In addition, hydrogen bonding is observed with the methoxy phenyl group of CMO and Gln247 and Lys218 of p65. The results of *in silico* analysis are in agreement with the results of cell based assays.

**Table 2:** Molecular docking results of p65 with oxadiazoles.

Entry	LS1	LS2	-PLP1	-PLP2	JAIN	-PMF	DS
1	2.26	5.49	91.7	82.35	0.23	54.81	54.86
2	2.19	5.31	90.55	83.5	0.37	52.69	53.72
3	2.23	5.40	91.14	85.13	0.34	53.51	53.98
4	2.96	5.94	86.94	81.35	2.02	51.45	56.80
5	3.60	5.68	95.92	85.44	0.09	56.92	58.38
6	2.70	5.72	78.03	71.04	0.46	48.53	51.54
7	2.70	5.47	77.54	71.31	0.35	50.87	50.72
8	2.42	5.59	78.85	74.85	0.48	50.49	52.58
9	5.29	5.77	80.3	75.22	1.6	29.64	56.64
10	4.00	6.26	84.55	74.9	2.85	46.21	61.00

*LS1 and LS2: LigScore1 and 2 are a fast, simple, scoring function for predicting protein–ligand binding affinities*.

*PLP1 and PLP2, piecewise linear potentials 1 and 2 are fast, simple, docking function that has been shown to correlate well with protein–ligand binding affinities*.

*JAIN, an empirical scoring function (lipophilic, polar attractive, and polar repulsive interactions, solvation of the protein and ligand, and an entropy term for the ligand) through an evaluation of the structures and binding affinities of a series of protein–ligand complexes*.

*PMF, potential of mean force is the scoring function developed based on statistical analysis of the 3D structures of protein–ligand complexes*.

*DS, Dock Score, ligand poses are evaluated and prioritized according to the Dock Score function*.

## Conclusion

In summary, this study aimed at designing a library of chemically novel and biologically active 1,3,4-oxadiazoles. We generated 10 oxadiazole structural analogs and evaluated for their cytotoxic effect against HCC cells, and the lead compound (CMO) displayed good antiproliferative efficacy. Based on the literature, we speculated NF-κB signaling as the putative target of the lead compound, and it is validated *via in vitro* and *in silico* approaches. Although, this study identifies NF-κB signaling as the likely target of 1,3,4-oxadiazoles, more comprehensive study on its off-targets, signaling cross talks and *in vivo* antitumor potential needs to be investigated.

## Author Contributions

KR, B, CM, AC, SA, AK, and GS conceived the project. KR, B, CM, GS, and AB designed the experiments. NA, SR, B, MS, and SM carried out the research and analysis of data. KR, B, AK, GS, CM, and NA wrote the paper.

## Conflict of Interest Statement

The authors declare that the research was conducted in the absence of any commercial or financial relationships that could be construed as a potential conflict of interest.
