# Supplementary material for: Novel 1,3,4-Oxadiazole Induces Anticancer Activity by Targeting NF-κB in Hepatocellular Carcinoma Cells
Source: Front Oncol. 2018 Mar 19;8:42. doi: 10.3389/fonc.2018.00042 (PMC5867297; doi:10.3389/fonc.2018.00042)
Supplement: Supplementary file 1 [file data_sheet_1.DOCX]

**Novel 1, 3, 4-Oxadiazole Induces Anticancer Activity by Targeting NF-κB in Hepatocellular Carcinoma Cells**

**Supplementary Data**


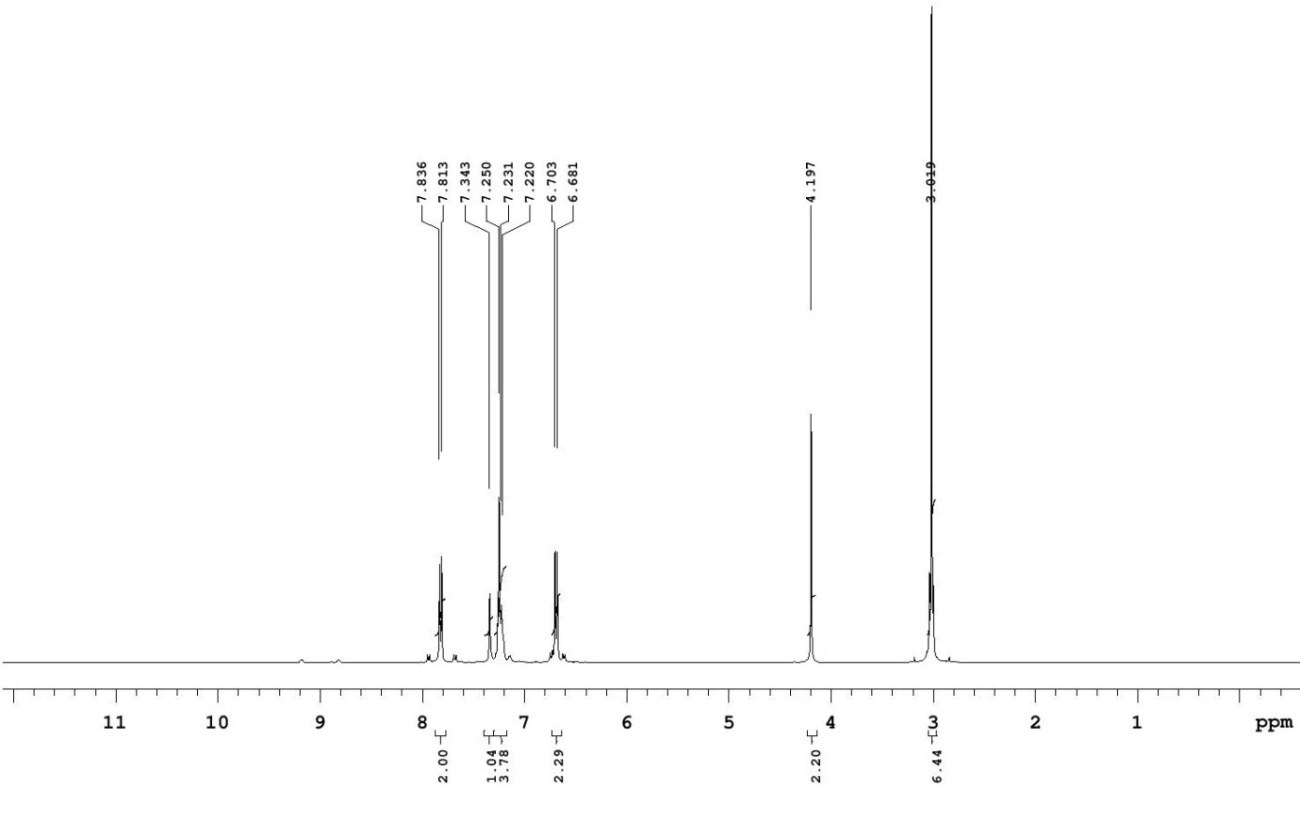


^1^H NMR (400 MHz) spectrum of compound **5a** in CDCl_3_.

**
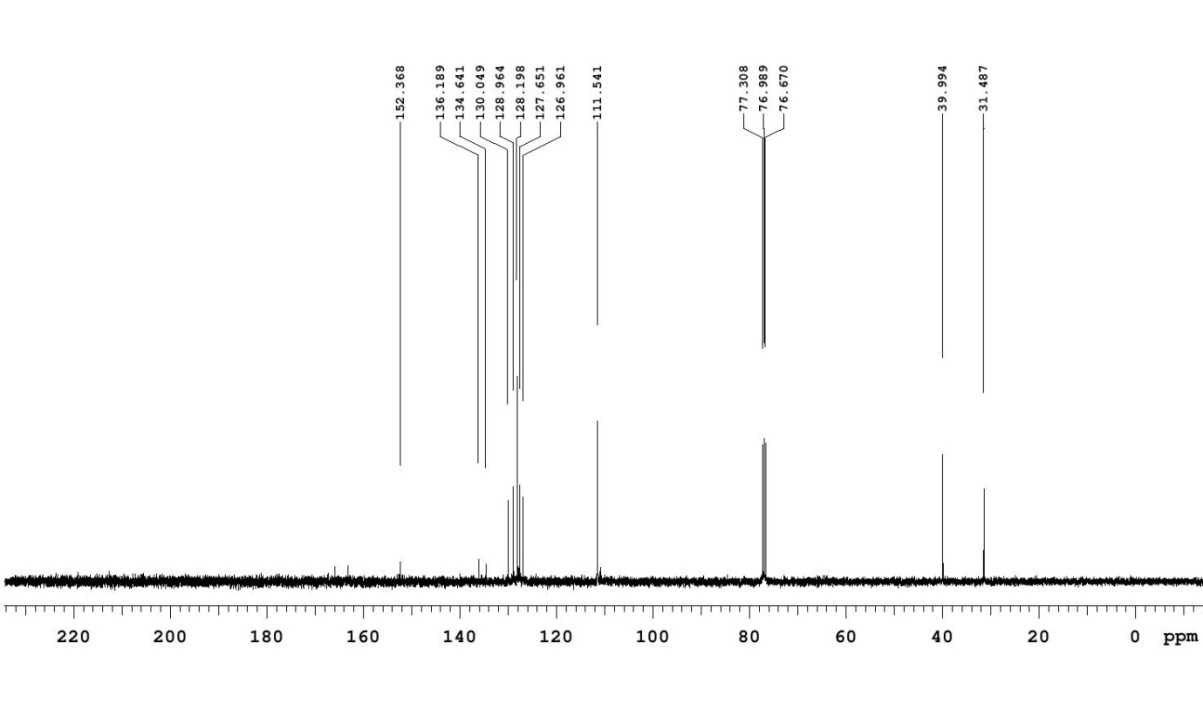
**

^13^C NMR (100MHz) spectrum of compound **5a** in CDCl_3_

**
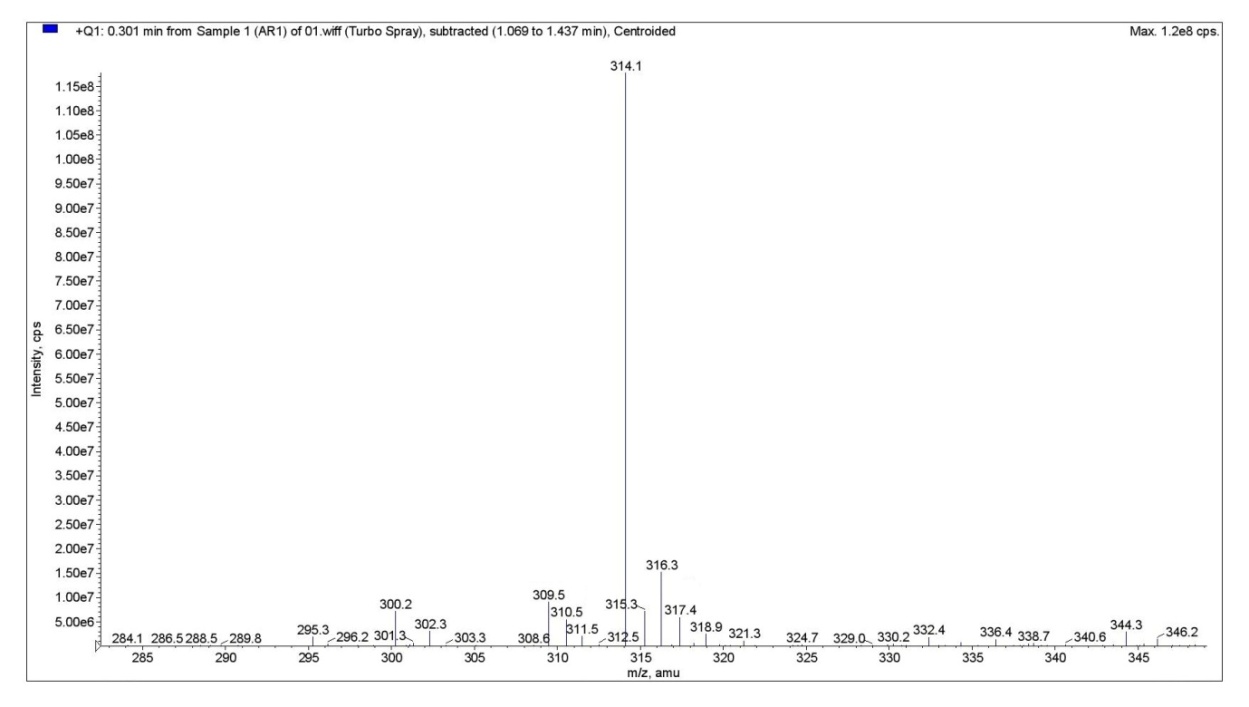
**

ESI Mass spectrum of compound **5a**

**
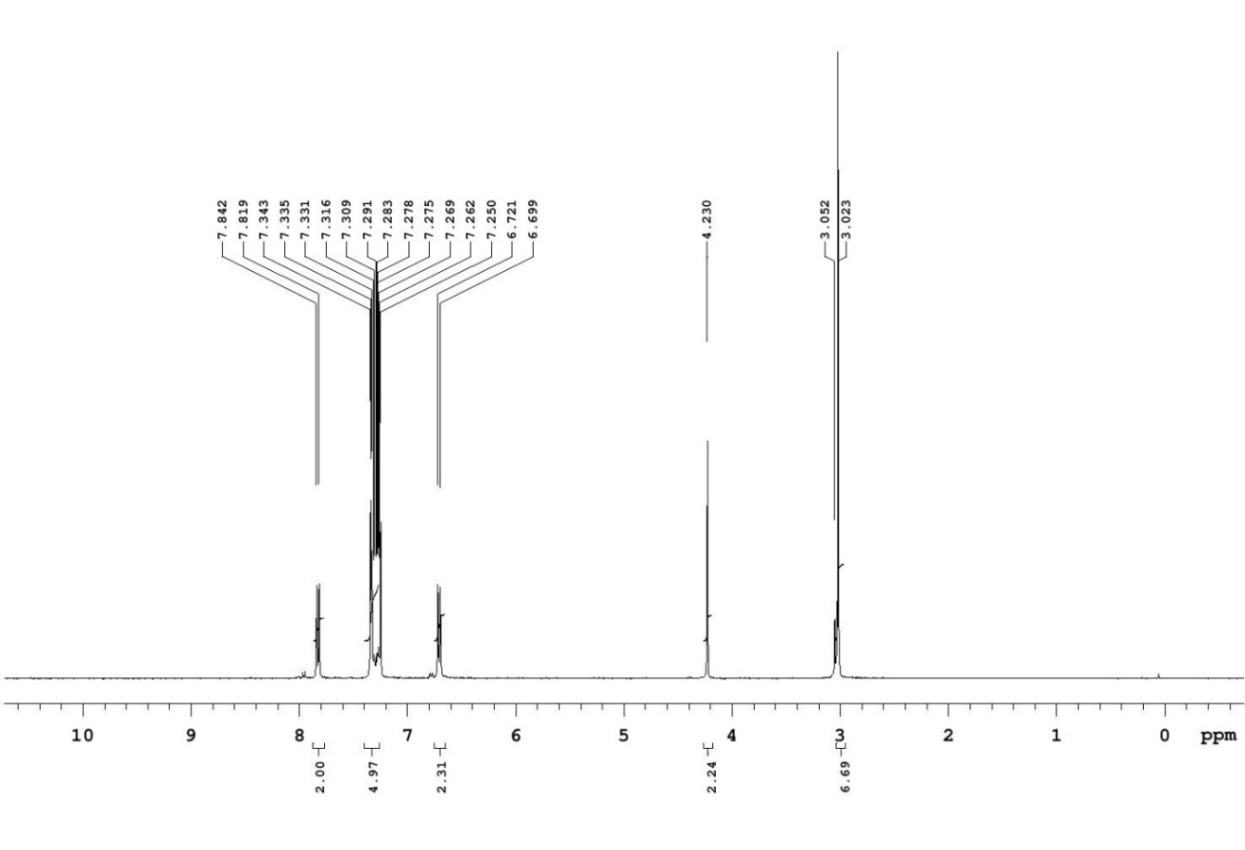
** ^1^H NMR (400MHz) spectrum of compound **5b** in CDCl_3_

**
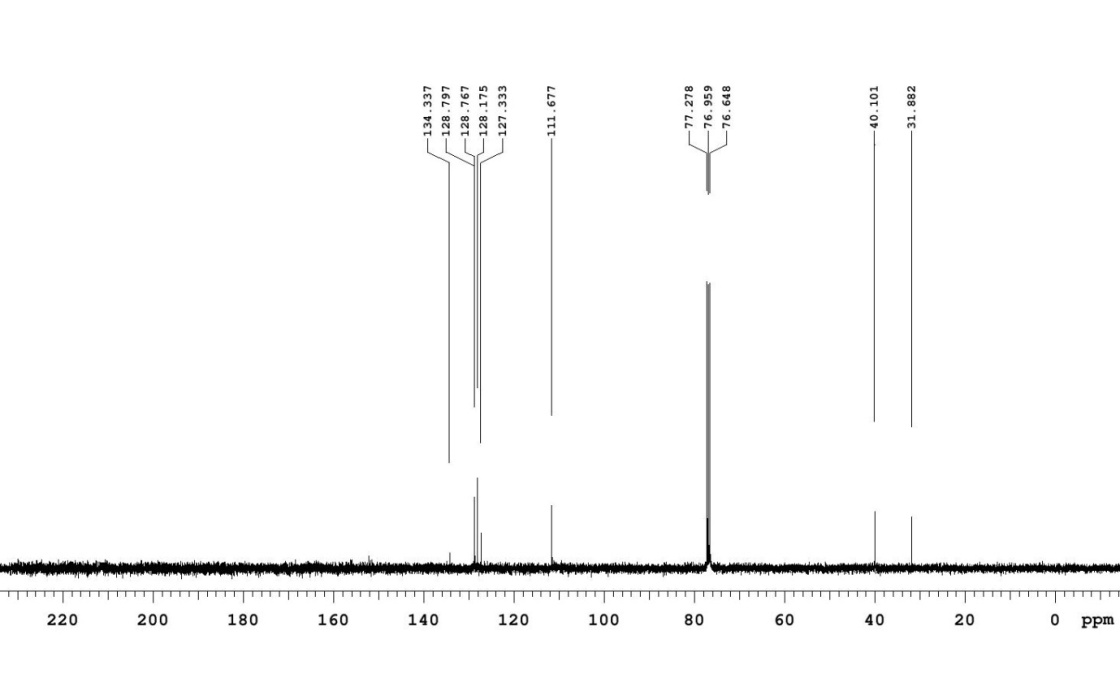
**

^13^C NMR (100 MHz) spectrum of compound **5b** in CDCl_3_

**
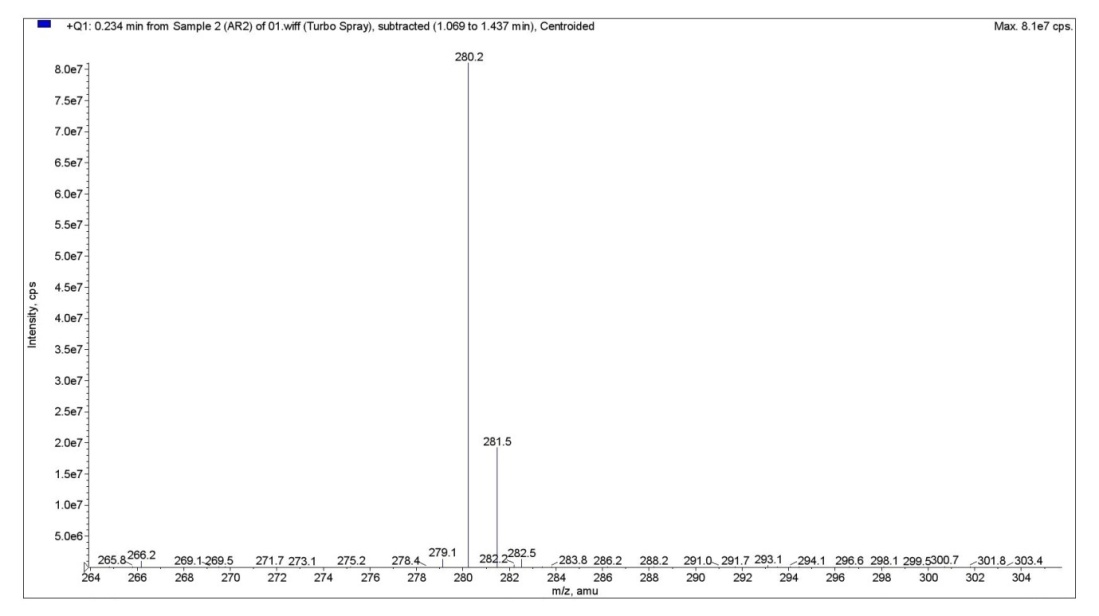
**

ESI Mass spectrum of compound **5b**

**
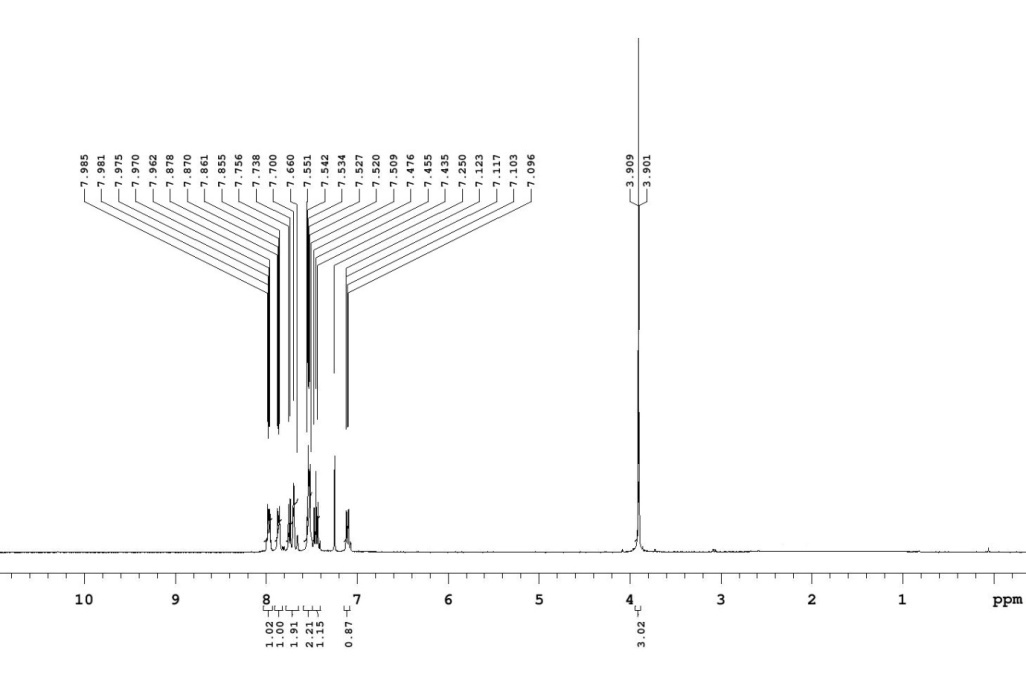
**

^1^H NMR (400MHz) spectrum of compound **5i** in CDCl_3_.

**
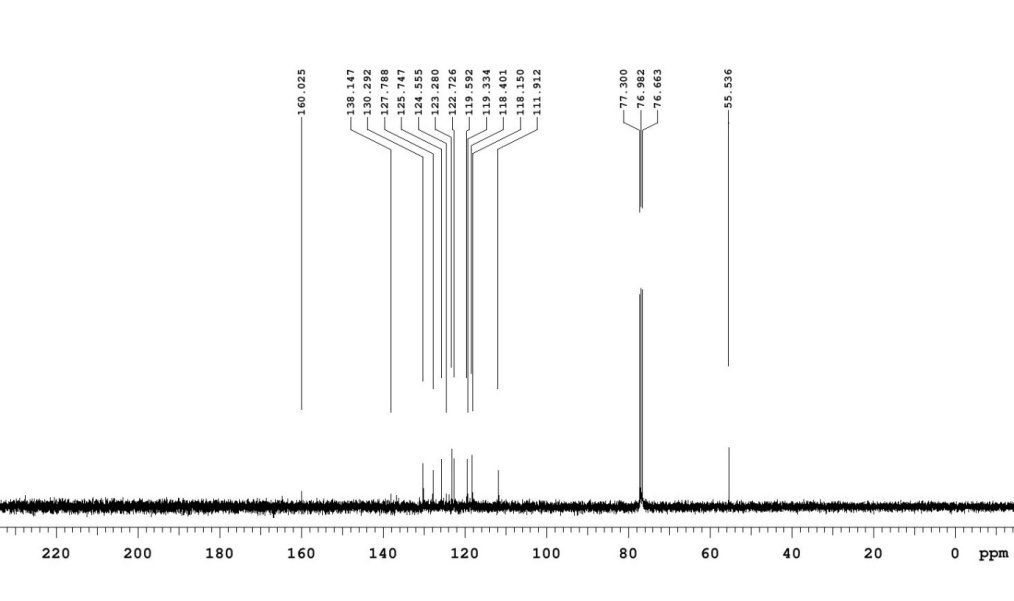
**

^13^C NMR (100MHz) spectrum of compound **5i** in CDCl_3_

**
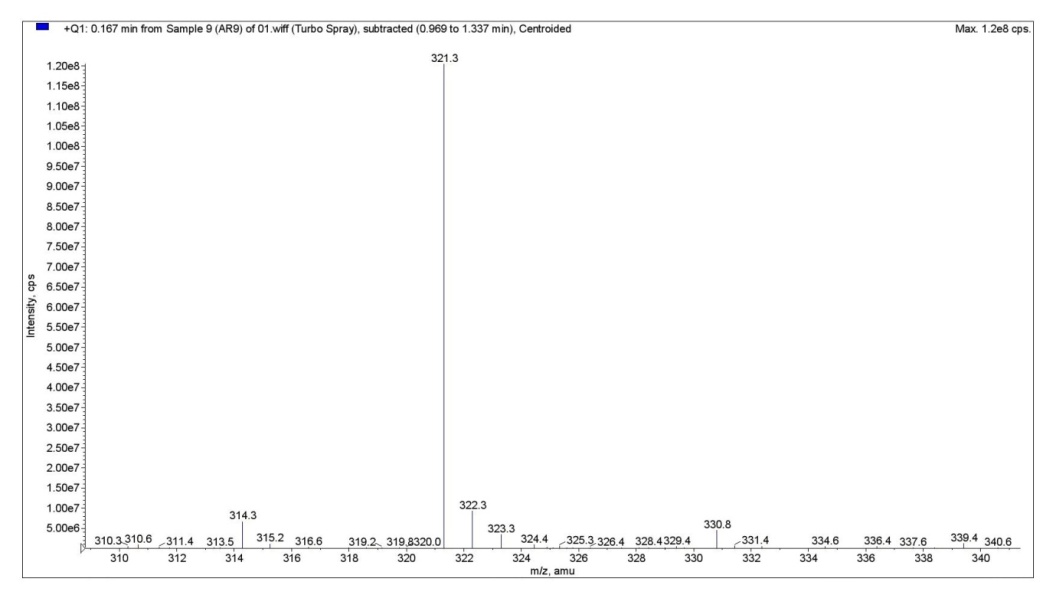
**

ESI Mass spectrum of compound **5i**
